# Investigation of Thermoluminescence Characteristics and Kinetics of Natural Obsidian

**DOI:** 10.1002/bio.70488

**Published:** 2026-04-19

**Authors:** Hamide Avci

**Affiliations:** ^1^ Department of Physics, Faculty of Sciences Selcuk University Konya Turkey

**Keywords:** anomalous heating rate effect, grain size effect, obsidian, thermoluminescence, TL dose response, TL kinetic analysis

## Abstract

This study investigates the thermoluminescence (TL) properties of natural obsidian samples collected from the Aksaray region in Turkey. The obsidian samples were ground into powder and sieved into five particle size ranges between 63 and 500 μm. The grain size range of 63–125 μm was identified as the most suitable for TL measurements, as it produced a sharper and more intense peak in the dosimetric region of the spectrum. Subsequently, detailed analyses were conducted on the TL glow curves, reusability, dose response, and heating rate behavior of the selected sample. The effect of heating rate was tested over a range of 0.1°C–10°C/s, revealing an anomalous heating rate dependence. Kinetic analysis using the $T_{m}-T_{\text{stop}}$ method identified multiple peaks, indicating the presence of various energy levels. TL curve deconvolution using the general order kinetics model yielded activation energies ranging from 0.82 to 1.68 eV and demonstrated a strong correlation between the experimental data and theoretical predictions.

## Graphical abstract

Thermoluminescence (TL) properties of natural obsidian from the Aksaray region were investigated under beta irradiation. The study identifies optimal grain size, evaluates kinetic parameters via *T*_*m*_–*T*_*stop*_ and computerized glow curve deconvolution (CGCD) methods, and demonstrates the material's potential for low‐dose environmental and personal radiation dosimetry.

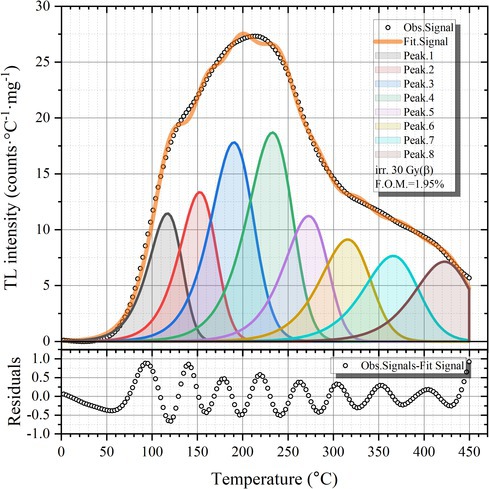

## 1 Introduction

Luminescence dosimetry represents an important component of solid‐state dosimetry, encompassing processes in which energy absorbed from ionizing radiation is subsequently emitted as light [1]. One of the key techniques within this field is thermoluminescence (TL). TL is used to evaluate the absorbed dose of ionizing radiation to which a phosphor is exposed, by analyzing the intensity of luminescence (i.e., the concentration of trapped charges) emitted from heated phosphors. TL methods utilize both natural and synthetic materials for dosimetric studies, including environmental, clinical, personal, and accident‐related assessments. Many natural minerals that are crucial for radiation research exhibit thermoluminescent properties influenced by their genesis, chemical composition, impurity content, and geological history [2]. For example, silicates (e.g., quartz and feldspar), carbonates (e.g., calcite and aragonite), phosphates (e.g., apatite and xenotime), and fluorides (e.g., fluorite) exhibit various TL behaviors [2, 3, 4]. Among these, quartz, feldspar, and calcite are particularly notable due to their strong thermoluminescent properties, making them potential candidates for applications in dosimetry and dating [5].

Obsidian is a naturally occurring volcanic glass formed from rapidly cooled lava. The material exhibits a physical state that is amorphous and isotropic, a characteristic attributable to its wholly disordered atomic arrangement. The principal component of obsidian is silica; however, it may also be intermingled with a variety of additional substances derived from the parent magma depending on the geographic origin of the material. Owing to the rich variety of minerals it contains, obsidian has found extensive use in alternative medicine, adjunctive therapy, spa treatments, cosmetics, and jewelry [6]. This stone is predominantly observed in hues of brown, black, or gray, and, with lesser frequency, in shades of green, blue, or red [7].

Numerous studies on obsidian can be found in the literature. Some authors have investigated the structural and radiation‐shielding properties of metal oxide‐doped glasses based on obsidian [8]. Their findings indicate that these obsidian‐based glass systems may serve as viable alternatives for industrial, medical, and technological applications. In addition, some obsidians have been found to be a good phosphor for dosimetry and have been recommended for use as dosimeters and for calibration of laboratory sources [9]. Moreover, various physical methods [10, 11, 12] and chemical methods [13, 14, 15, 16, 17] have been proposed to ascertain the provenance of obsidian. To effectively integrate obsidian into technological applications, multidisciplinary scientific research is required. However, there are very few publications about the luminescent properties of natural obsidian in the literature [6, 7, 9, 18, 19].

The aim of this study is to investigate the thermally stimulated luminescence (TL) properties of a natural obsidian sample and to evaluate its potential for radiation dosimetry. Turkey is particularly rich in obsidian reserves, and these natural materials are readily available and workable. However, there are very few articles in the literature on the TL properties of obsidian. Although some studies have reported general luminescent responses of obsidian, its TL kinetic parameters, trap distributions, and dosimetric performance have not been systematically analyzed. This study therefore addresses this gap by conducting a detailed TL kinetic analysis of natural obsidian, aiming to clarify its trap structure and evaluate its suitability for radiation dosimetry applications. The sample under investigation is natural obsidian, provided by the Konya Technical University Ornamental Stones Application and Research Center, and was sourced from Aksaray region. In this context, for the first time, the present study investigates the TL properties of natural obsidian samples collected from Aksaray, as previously mentioned.

## 2 Materials and Methods

**FIGURE 1:**
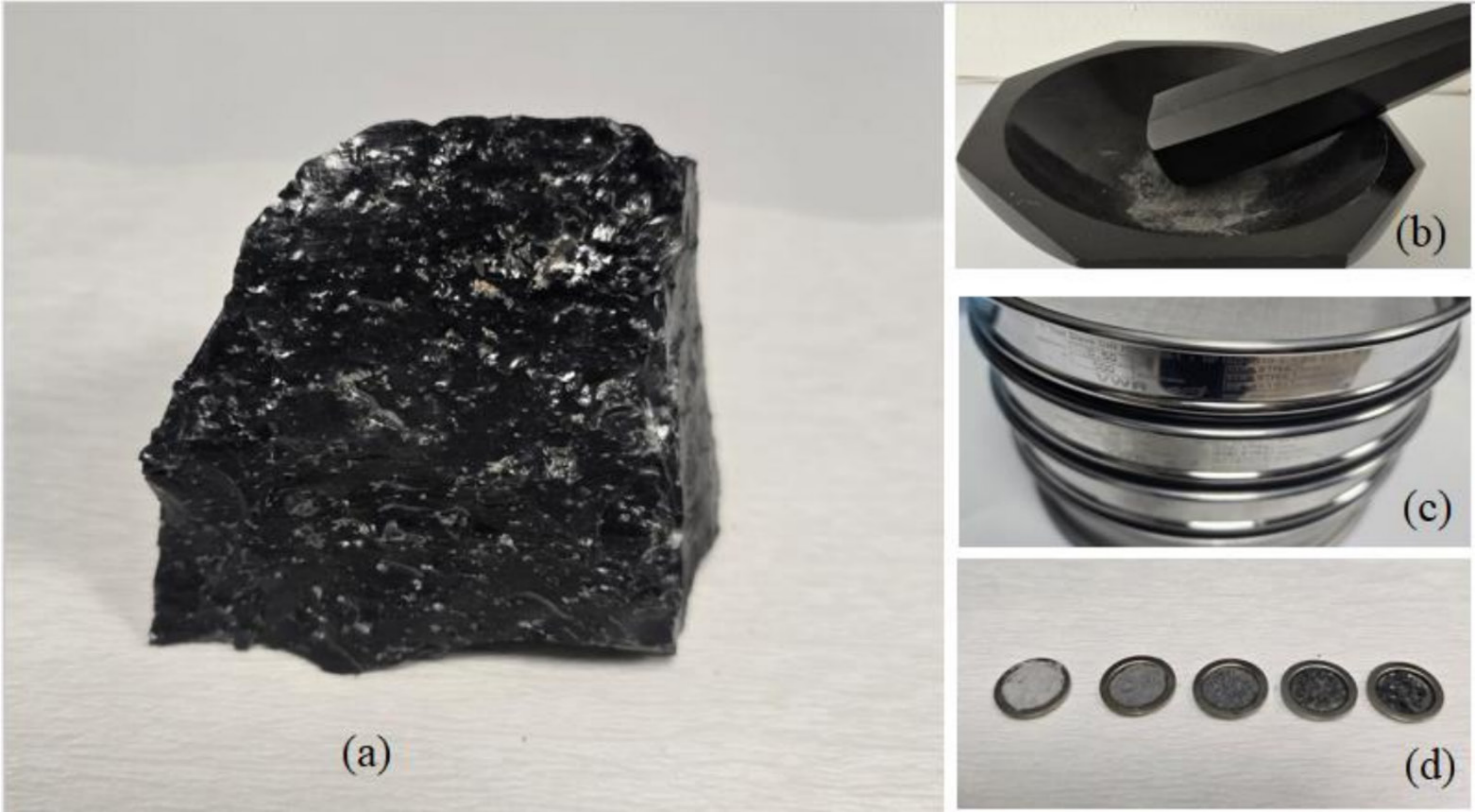
Aksaray Obsidian (a) in natural form, (b) powder, (c) in sieved form, and (d) in planchet.

The obsidian samples within the scope of this research were brought from Aksaray region of Turkey. The natural obsidian sample (Figure 1a) was manually ground using a mortar and pestle (Figure 1b). The resulting obsidian powder was subsequently sieved through five different mesh sizes, ranging from 63 to 500 μm, to achieve the desired particle size distribution (Figure 1c). The samples obtained through the sieving method resulted in powders with particle size fractions of less than 63, 63–125, 125–250, 250–500, and larger than 500 μm (Figure 1d). Three aliquots, which are in powder form, were prepared for each particle size. Even though the three aliquots exhibit nearly identical properties, they were obtained from the same chunk of material. No additional chemical or physical purification processes were applied to the obsidian samples prior to characterization. This approach was deliberately chosen to preserve the natural structural and compositional characteristics of the obsidian, thereby ensuring that TL responses accurately reflect the intrinsic properties of the material in its natural state. In this study, the samples were tested only using beta radiation with a low dose rate. X‐ray diffraction (XRD), scanning electron microscopy (SEM), and energy dispersive X‐ray spectroscopy (EDX), and Fourier transform infrared spectrometry (FT‐IR) analyses for the physical and chemical characterization of all particle sizes of powdered obsidian were carried out at Selçuk University Advanced Technology Research and Application Centre (ILTEK).

TL measurements were performed using the Risø TL/OSL reader (model TL/OSL‐DA 20) [20, 21], which is operated at the Luminescence Laboratory located at the Physics Department of Selçuk University and jointly managed by the Physics Department and Nuclear Materials Research and Application Center of Selçuk University. This reader contains an internally calibrated ^90^Sr/^90^Y beta radiation source with a dose rate of 0.0625 Gy/s as of the date when the measurements were conducted [22]. All subsequent procedures, including irradiation, and TL measurements, were conducted under laboratory conditions.

The process of acquiring TL spectrum readings can be enhanced through the implementation of optical bandpass filters positioned between the sample designated for measurement and the photomultiplier tube utilized for the observation of the phenomena exhibited by the dosimetric phosphorus material. A TL spectrum encompasses the aggregation of all wavelengths within the visible spectrum, enabling the meticulous analysis and observation of the responsive characteristics of the material under investigation, particularly regarding TL intensity, TL peak position, and related parameters. This process uses two separate bandpass filters: the Hoya U340 (2.5 mm) filter, which is sensitive to emission in the ~340 nm region, and a combination of Schott BG 3 (1 mm) and Schott BG‐39 (1 mm) filters, which are sensitive to emission in the ~410‐nm region. Firstly, TL readings were taken to decide which filter to use, and the results were evaluated for signal quality and reliability. The particle size with the most intense TL signal and the least deviation from the initial reading was determined for different sizes of obsidian. All subsequent TL experiments were performed using the selected particle size sample. All the TL measurements were performed at a linear heating rate of 5°C s^−1^ from room temperature up to 400°C in a nitrogen atmosphere (except for analyzing various heating rates experiment (VHR)). In TL characterization experiments, the selected particle size sample was exposed to beta radiation at various dose levels to determine its dose‐dependent properties such as radiation sensitivity range and linearity. The sensitivity range is essential for determining the potential applications of the dosimetric material, including personnel monitoring, retrospective dosimetry, and accident assessment. As well as the effect of heating rates on the TL glow, curve properties and repeatability testing were also investigated. Additionally, the complex TL glow curve was examined for individual peaks by analyzing data obtained from the $T_{m}-T_{\text{stop}}$ experiments [23]. The TL analyses were finalized with a computerized glow curve deconvolution (CGCD) analysis, which is proposed as the theoretical framework for the $T_{m}-T_{\text{stop}}$ experiment.

## 3 Results and Discussion

### 3.1 XRD

**FIGURE 2:**
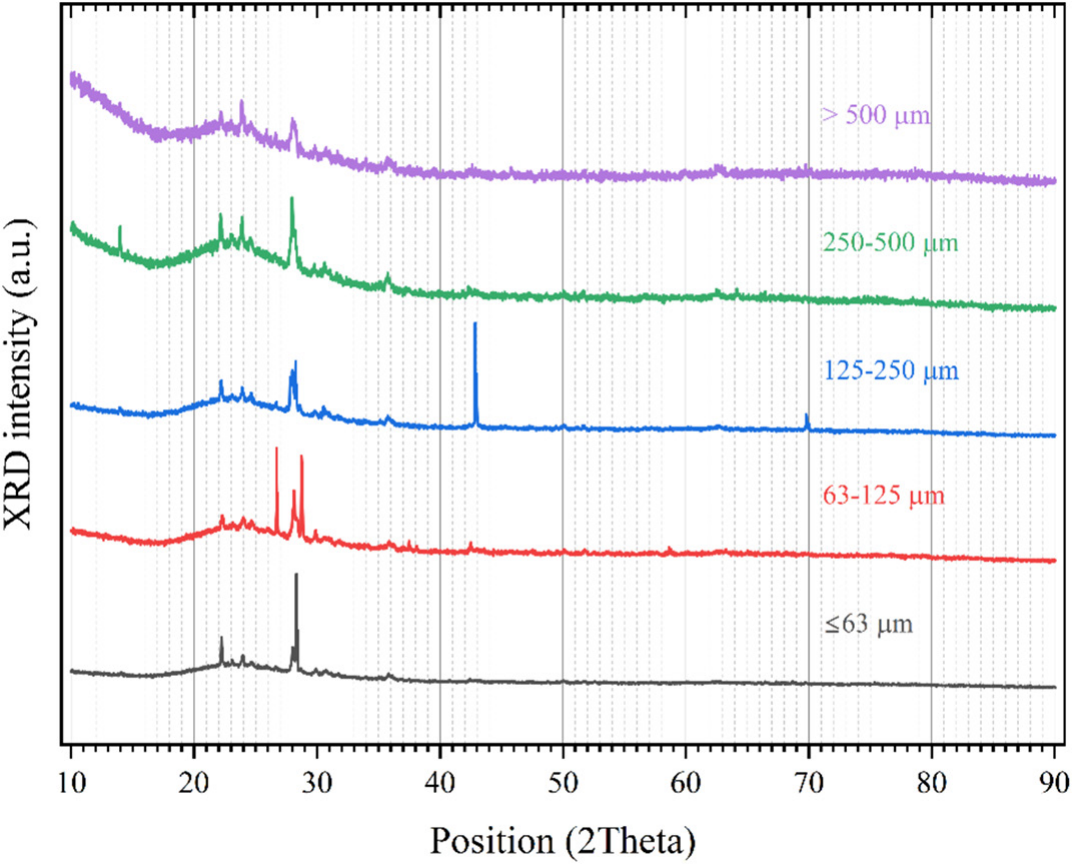
Experimental X‐ray diffraction patterns for different grain size groups of Aksaray Obsidian.

The XRD pattern analysis results for all particle sizes of obsidian powder samples are shown in Figure 2. The XRD pattern obtained for the powdered obsidian (Figure 2) exhibits broadbands, characteristic of amorphous materials that lack long‐range order [24, 25].

### 3.2 SEM

**FIGURE 3:**
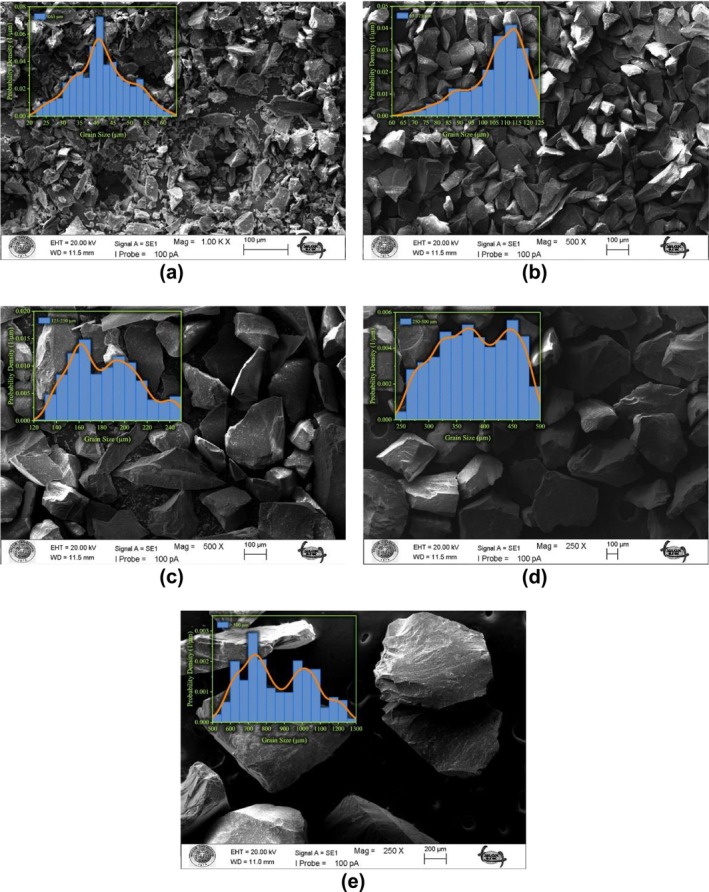
SEM images of obsidian samples particle size fractions of (a) equal and less than 63 μm, (b) 63–125 μm, (c) 125–250 μm, (d) 250–500 μm, (e) and larger than 500 μm. Grain size distribution histograms are shown as insets within each image.

The microstructure of all particle size fractions of obsidian powders was analyzed using SEM. Prior to SEM measurements, the powdered samples were coated with a thin layer of gold to enhance conductivity. Figure 3 presents SEM images of obsidian particles sorted by size: (a) ≤ 63 μm, (b) 63–125 μm, (c) 125–250 μm, (d) 250–500 μm, and (e) > 500 μm. The images reveal irregular but generally flattened grain surfaces, characteristic of the glassy texture of obsidian. These morphological features are consistent with previous findings that identify obsidian as a predominantly silicate‐based material [26].

Inset within each SEM image is a histogram showing the corresponding grain size distribution, based on measurements performed using open‐source ImageJ software [27]. The histograms display probability density functions derived from the measured grain sizes. A kernel density estimation (KDE) method was applied to fit a smooth curve to the data, providing a continuous representation of the distribution. The *x*‐axis of each histogram denotes grain size in micrometers (μm), while the *y*‐axis represents probability density, with units of 1/μm. The KDE curve is normalized such that the area under the curve equals 1, enabling interpretation of the data as a probability distribution. This method offers a more detailed and accurate visualization of grain size variation compared to conventional histogram binning.

### 3.3 EDX

To ascertain the chemical composition of all obsidian samples, EDX analyses were performed. The results are summarized in Table 1, which details the chemical composition of the examined samples, obtained through microanalysis, and is characteristic of natural obsidian. This analysis reveals a composition predominantly consisting of SiO_2_, indicative of a significant presence of amorphous silica, alongside various other elements such as Al, K, Na, Fe, Ca, Mg, Mn, and Ba.

**TABLE 1:** Chemical composition (%) of the obsidian samples all of particle sizes by the EDX measurements.

Grain size	Chemical element (weight [%])									
	Si	O	Al	K	Na	Fe	Ca	Mg	Mn	Ba
≤ 63	23.49	65.54	5.66	1.76	2.23	0.48	0.64	0.09	0.06	0.06
63–125	23.61	65.17	5.53	2.11	2.39	0.62	0.49	0.08	0.00	0.00
125–250	22.98	65.69	5.54	1.95	2.63	0.51	0.45	0.14	0.05	0.07
250–500	23.91	63.38	6.13	1.80	3.46	0.47	0.39	0.37	0.02	0.05
> 500	20.57	69.96	4.79	1.45	2.25	0.51	0.24	0.12	0.07	0.03

### 3.4 FT‐IR

**FIGURE 4:**
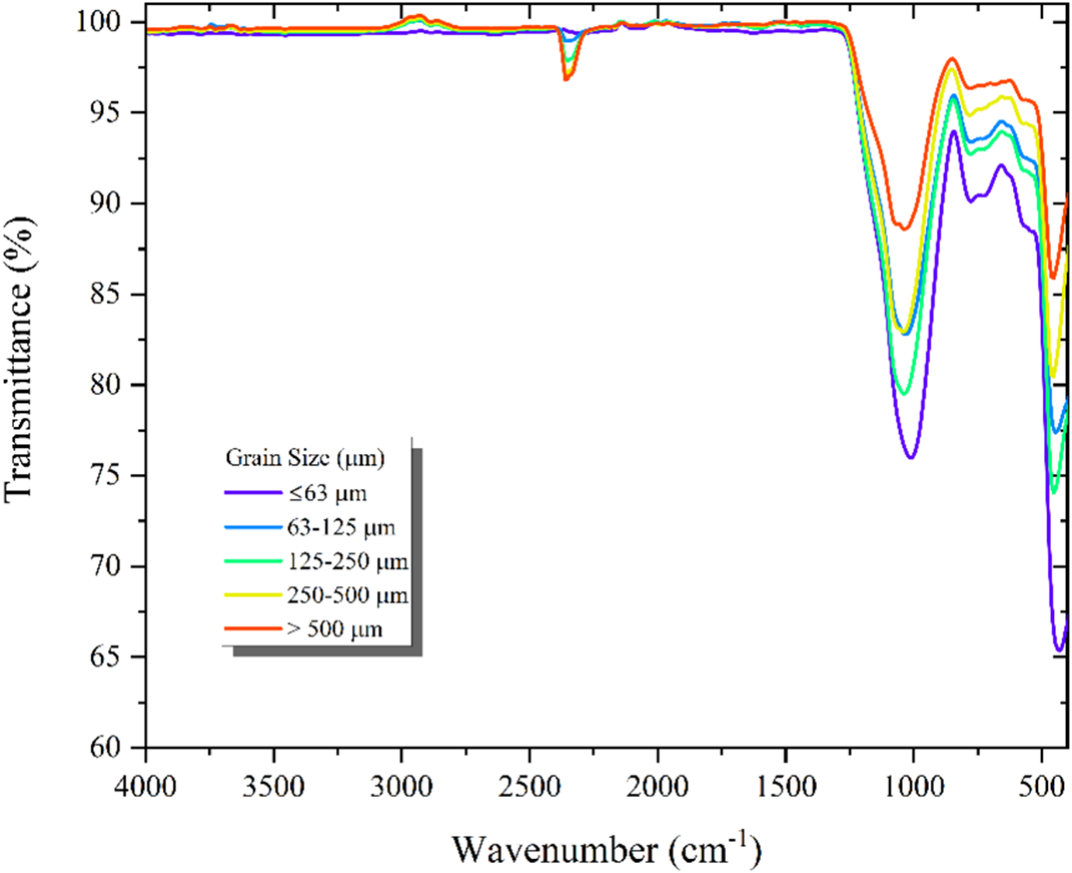
FT‐IR spectra recorded of obsidian samples.

FT‐IR analysis was performed to examine the chemical structure of all particle size fractions of obsidian powders. The analysis was carried out in the wavelength range of 400–4000 cm^−1^, and the transmittance spectrum is given in Figure 4. Generally, with the exception of the intensities, all samples exhibit similar properties in the range of approximately 400–2500 cm^−1^. The intense peak observed at 1036 cm^−1^ in the FT‐IR spectra represents the Si‐O‐Si and Si‐O stretching peaks. The Si‐O‐Si peak is particularly intense and broad, suggesting that these peaks are combined. The moderately intense peak observed at 781 cm^−1^ is the Si‐O stretching peak [28, 29]. The weak peak observed at 680 cm^−1^ indicates Si‐Si bonding [30]. It is also observed that the Al‐O stretching originating from the Al_2_O_3_ present in the material contributes to the peaks observed at 781 and 680 cm^−1^. The peaks observed at 580 cm^−1^ indicate the Fe‐O peak. However, this peak was observed weakly due to the dominance of SiO_2_ in the structure. The peak observed at 458 cm^−1^ originates from the Fe‐O‐Fe and Si‐O stretching. In particular, when the material was tested with a strong magnet, it was observed that the material exhibited magnetic properties and oriented toward the magnet, supporting the observed Fe‐O‐Fe peak. This indicates the presence of the Fe_3_O_4_ structure in the material [29, 31, 32]. Generally, FT‐IR analysis supports the finding that the material contains SiO_2_, Al_2_O_3_, and Fe_3_O_4_.

### 3.5 TL Glow Curve Characteristics

**FIGURE 5:**
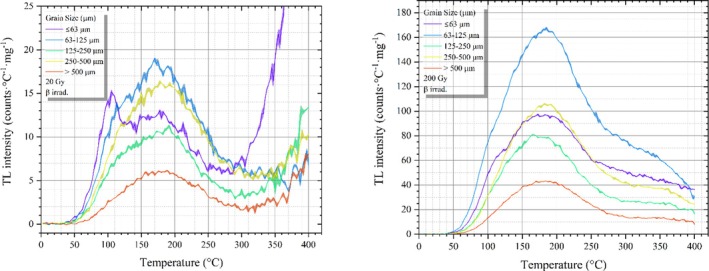
Net TL signals were recorded for all grain size groups after exposure to 20 and 200 Gy of beta radiation.

In this study, TL emission was detected through a blue filter package: a combination of Schott‐ BG3 and Schott‐BG39 filters. A background signal reading was performed continuously before and after each TL signal reading. The background signal was then subtracted from the TL signal to obtain the net TL signal. Thus, temperature‐based background correction was performed. The net TL signal data were used in the analysis of the experiments conducted in this study. All samples were irradiated with beta particles at a dose level of 20 and 200 Gy for the purpose of detecting thermoluminescent (TL) emission. TL spectra were recorded using a linear heating rate of 5°C/s from 0°C to 400°C. This temperature limit was selected to preserve the natural trap structure and the associated luminescence properties of the obsidian, as also discussed in the literature [7, 33]. As can be seen in Figure 5, the TL spectrums of the obsidian samples exhibit a peak around ~180°C. To see the effect of particle size, the results of TL measurements for each particle size of obsidian samples irradiated with 20 and 200 Gy beta radiation were prepared and shown in Figure 5. The increase in particle size changes the TL sensitivity of the material as the particle size increases above the 63‐ to 125‐μm range. The sensitivity also decreased for particles smaller than this size range, indicating that the material in this particular particle size range exhibited the highest sensitivity. Therefore, particle range of 63–125 μm was chosen, and the rest of the TL studies were performed in this size range.

In comparing my study with existing literature, the TL radiation curves derived from the examined samples display peak structures and thermal stability characteristics that align with those reported in previous studies, such as the primary peak observed around 220°C in the study of P.L. Antonio et al. (2019) [7] and around 270°C in the study of I. Liritzis and Strofilas (2010) [18]. Furthermore, the current study contributes to a more in‐depth understanding of the trap structure and TL behavior of natural obsidians through the use of the $T_{m}-T_{\text{stop}}$ and CGCD methods.

### 3.6 Repeatability of TL Response

**FIGURE 6:**
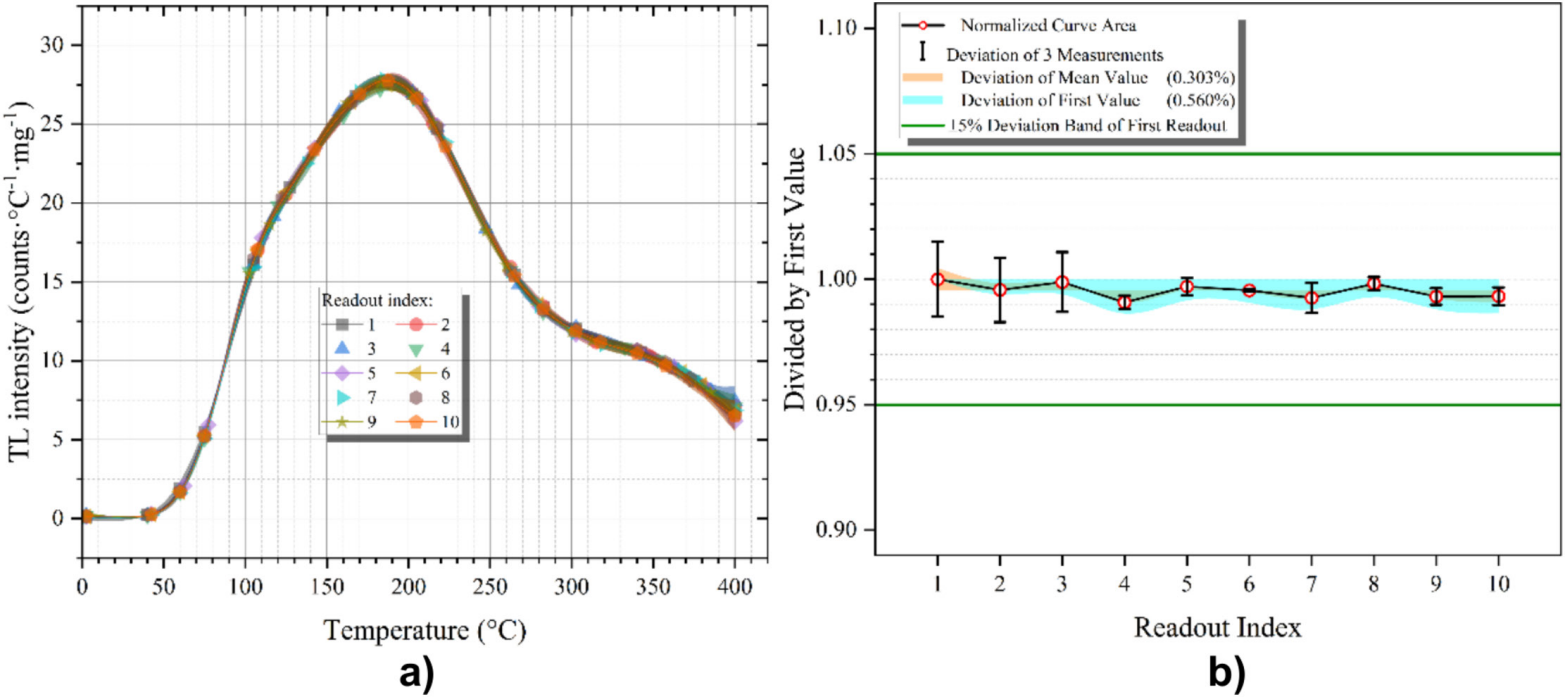
TL repeatability trend of the obsidian sample with a grain size 63–125 μm after irradiation with beta source (30 Gy).

The repeatability of good dosimetric material should have a standardized structure of less than 5% of repeated measurements under the same dose and reading conditions [1, 34]. The reliability of the response exhibited by the selected obsidian samples (63–125 μm) was substantiated through the execution of a reproducibility investigation. The obsidian sample was irradiated with a beta dose of 30 Gy, and then TL reading was performed. This procedure was repeated across 10 measurement cycles, and the entire experiment was performed in three independent sets. The results are shown in Figure 6a. Figure 6b shows the normalized sum of net TL signals (curve area) obtained from these three sets. The TL signals were normalized to the first TL readout of each set. As shown in Figure 6b, the obtained standard deviations of the normalized integrated TL intensity are less than 5%. It is clearly seen that the result is within the specified tolerance ranges.

### 3.7 Dose Response Curves and Lower Detection Limit (LDL)

**FIGURE 7:**
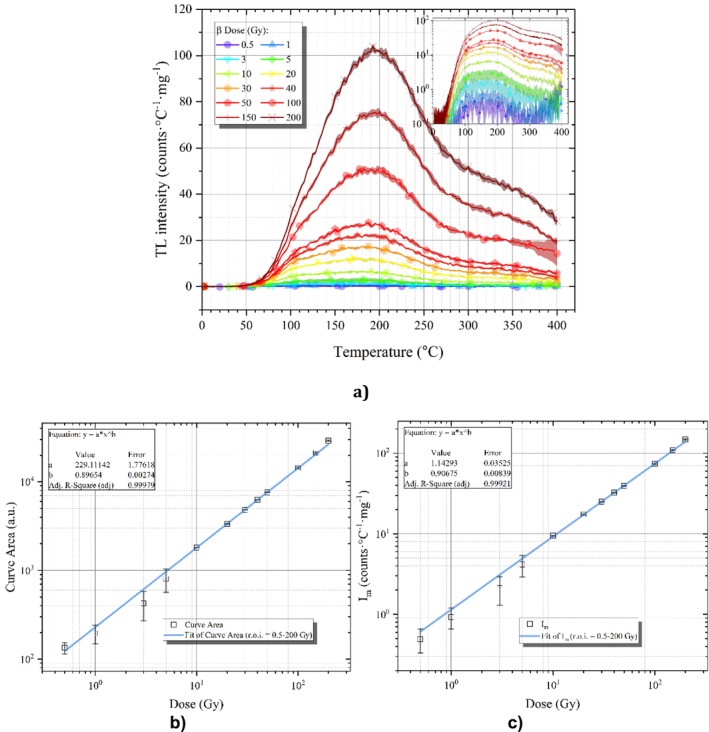
(a) Dose response behavior of the TL glow curves. (b) Dose response of obsidian sample according to the area under TL glow curve. (c) Dose response of the obsidian sample according to the TL intensity.

Evaluating the dose sensitivity of any candidate thermoluminescent dosimeter (TLD) material through TL experiments is essential for establishing its potential applications [35]. The sample's TL signal was measured following exposure from 0.5 to 200 Gy, with varying levels of beta irradiation for this purpose. For dose levels between 0.5 and 200 Gy, the TL reading process was repeated three times for each dose level. Figure 7a shows the results of varying dose responses. In the examination of visible maxima, it is observed that there is no substantial alteration in the morphology of the glow curve; however, it is evident that the peak temperatures migrate toward the higher temperature domain in conjunction with the escalation of the dose. The empirical findings derived from the dose–response curve yield foundational insights into the kinetic order. In accordance with TL theory, the peak temperature associated with a second‐order peak is anticipated to diminish with increasing dose, whereas the peak temperature corresponding to a first‐order peak is posited to remain unaffected by the dose [36]. In the current case, the visible maximum remains largely unchanged, exhibiting only a negligible variation of ± 5°C prior to the onset of saturation phenomena induced by high doses, which is characteristic of first‐order kinetics. However, the glow curve may not contain isolated TL peaks; the potential exists for overlapping peaks with different TL properties.

The dose–response linearity of any TL sensitive material can be assessed by constructing a log–log plot of either the TL peak intensity or the TL peak area against the applied dose. This distribution can then be fitted using a logarithmically scaled line function as described in Equation (1):

(1) $$I_{m}=aD^{b}$$

where $I_{m}$ Equation (1) is the TL peak intensity, *D* is the irradiation dose, *a* is where distribution intercept *y*‐axis, and b is the slope. If b=1 means dose response character of that material in that dose range is linear, for the b>1 is called “superlinear (or supralinear)” and b<1 is called “sublinear” [37]. Figure 7b,c illustrates the dose–response data, presenting a log–log scaled plot of the TL curve area and intensity in relation to doses ranging from 0.5 to 200 Gy. The slope (b) value obtained from the curve fitting Figure 7b is 0.89654 (*R* ^2^ = 0.99979) in the dose region between 0.5 and 200 Gy, while those obtained from Figure 7c is 0. 90,675 (*R* ^2^ = 0.99921) in the same dose region. Consequently, the glow peak exhibited sublinear range in the dose region between 0.5 and 200 Gy when the entire glow curve was examined according to Figure 7b,c.

Also, the LDL was calculated using Equation (2):

(2) $$\mathrm{LDL}=3\sigma_{\mathrm{bkg}}\frac{D}{M}$$

where $\sigma_{\mathrm{bkg}}$ denotes the standard deviation of three background signals obtained from nonirradiated readings, while D/M represents the calibration factor, calculated by dividing the dose value by the mean net TL signal obtained from three measurements conducted under identical experimental conditions [37, 38]. LDL for this obsidian sample obtained from Aksaray region was calculated as 71.85 ± 27 mGy. This value obtained is acceptable for low dose dosimetry.

### 3.8 Heating Rate Behavior

The heating rate is one of the important parameters affecting the position and shape of the TL radiation curve. The rate of heating during TL measurements is known to exert significant influences on the resultant TL glow curves, which manifest as the values of $T_{m}$ exhibit a tendency to migrate toward elevated temperature domains concurrently with the diminishment of the maxima heights, denoted as $I_{m}$ values, as the heating rate experiences an increase the total emission remains relatively constant (± 5%) [39].

**FIGURE 8:**
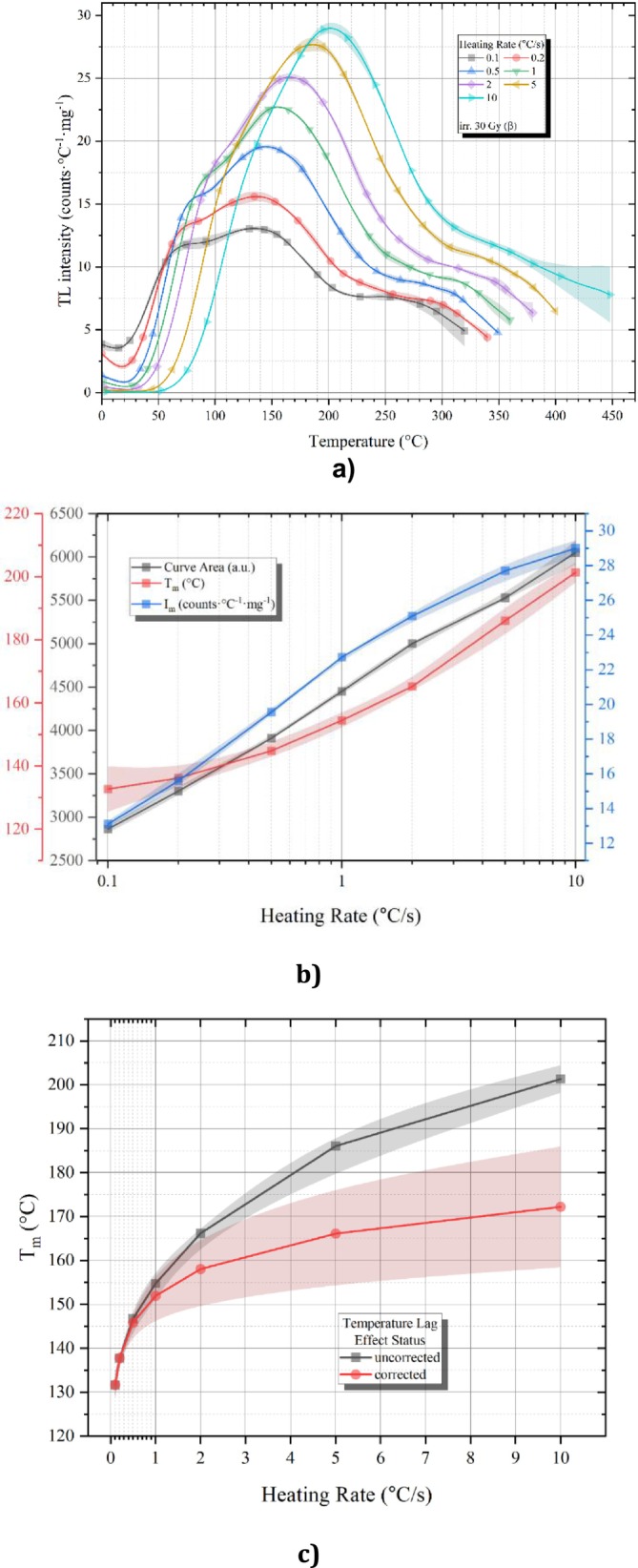
(a) TL glow curve emission curves obtained using variable heating rates for obsidian sample. (b) Heating rate trend for TL glow curve area, TL peak temperature, and TL peak intensity. (c) $T_{m}$ trend before and after TLA correction for obsidian sample.

In order to observe the heating rate effect, after the powdered obsidian sample was exposed to a β dose level of 30 Gy, the TL readings were completed by varying the heating rate from 0.1°C to 10°C/s. The reading process was repeated three times for each value of the heating rate. TL glow curves of the obsidian sample in Figure 8a were obtained using heating rates of 0.1°C, 0.2°C, 0.5°C, 1°C, 2°C, and 10°C/s. Consistent with expectations, the TL spectrum for the obsidian sample shows a shift toward higher temperatures. Additionally, as the heating rate increased, the TL density also increased. Consequently, an anomalous heating rate effect is seen in Figure 8a, which can be characterized as an increased TL intensity gain [40]. The peak area also increased as a result of this anomalous heating rate effect, as illustrated in Figure 8b, which presents the relationship between TL peak intensity ($I_{m}$), TL glow curve area, and TL peak maximum ($T_{m}$) in relation to the applied TL heating rates. It is essential to highlight that the TL glow curves were truncated, as previously mentioned in the manuscript, which introduces additional uncertainty in the calculation of the glow curve area. Nevertheless, the observed increase in TL intensity serves as a more reliable and convincing indicator of the anomalous heating rate effect.

The anomalous heating rate effect observed in the TL glow curves can be interpreted within the framework of the semilocalized transition model [41]. According to this model, an additional nonradiative transition occurs, involving the transfer of charge carriers from a localized excited state to a recombination center [42, 43]. The formation of the TL glow curve thus includes both localized transitions from the excited state to the recombination center and delocalized transitions from the conduction band to the same center [44]. This nonradiative transition induces an anomalous heating rate, resulting in a more intense TL glow. It is also responsible for the slower decay of the TL glow curve at elevated temperatures. As the heating rate increases, the probability of transitions from the localized excited state to the conduction band also rises. Importantly, nonradiative transitions remain unaffected by temperature, creating an interesting situation where the potential for radiative recombination increases with rising temperatures. This complex interaction ultimately contributes to the observed enhancement in TL peak intensity at higher heating rates. Figure 8a can be explained using a similar approach. As previously noted, the TL glow curves of obsidian samples exhibited a gradual shift toward higher temperatures. A potential explanation for this observed pattern is the temperature lag effect (TLE) [45].

The TL reader records the temperature from its heater; however, thermal conduction may be impeded by the contact between the sample cup and the sample, as well as the thermal conductivity properties of the materials. This phenomenon, referred to as TLE, influences the temperature value of the peak and can be corrected using Equation (3) [37, 45].

(3) $$T_{m_{j}}=T_{m_{i}}-\frac{T_{m_{2}}-T_{m_{1}}}{\ln\left(\frac{\beta_{2}}{\beta_{1}}\right)}\ln\left(\frac{\beta_{i}}{\beta_{j}}\right)$$

where $T_{m_{j}}$ and $T_{m_{i}}$ are the temperature maximum values obtained when applying heating rates $\beta_{i}$ and $\beta_{j}$, respectively. $\beta_{1}$ and $\beta_{2}$ are the slowest two heating rates among the applied ones. Consequently, both the corrected and uncorrected $T_{m}$ values are presented in Figure 8c along with their deviation as band. The corrected and uncorrected versions of the $T_{m}$ values calculated using Equation (3) are given in Table 2.

**TABLE 2:** TL peak temperatures against the heating rates before and after temperature lag correction applied.

Heating rate (°C/s)	*T*_*m*_ uncorr. (°C)	*T*_*m*_ corr. (°C)	∆T (°C)
0.1	131.7 ± 3.1	131.7 ± 3.1	0
0.2	137.8 ± 0.56	137.8 ± 0.56	0
0.5	146.81 ± 2.87	145.87 ± 2.8	0.94 ± 0.9
1	154.78 ± 3.15	151.97 ± 5.34	2.81 ± 2.31
2	166.21 ± 1.64	158.07 ± 7.87	8.14 ± 6.96
5	186.06 ± 4.98	166.14 ± 11.23	19.93 ± 7.43
10	201.35 ± 3.13	172.24 ± 13.76	29.12 ± 16.49

### 3.9 $T_{m}-T_{\text{stop}}$ Analysis and TL Glow Curve Deconvolution

Kinetic parameters refer to the specific characteristics related to defects within materials and serve as essential tools for elucidating the mechanisms of luminescence in various substances. Among these parameters that can be derived from the analysis of TL glow curve data are activation energy and the order of kinetics. A comprehensive examination of methodologies applicable for the extraction of initial rise, peak shape, variable heating rate, whole glow peak, and glow curve deconvolution, and so on, parameters from glow curve data are provided by Chen and McKeever [36].

**FIGURE 9:**
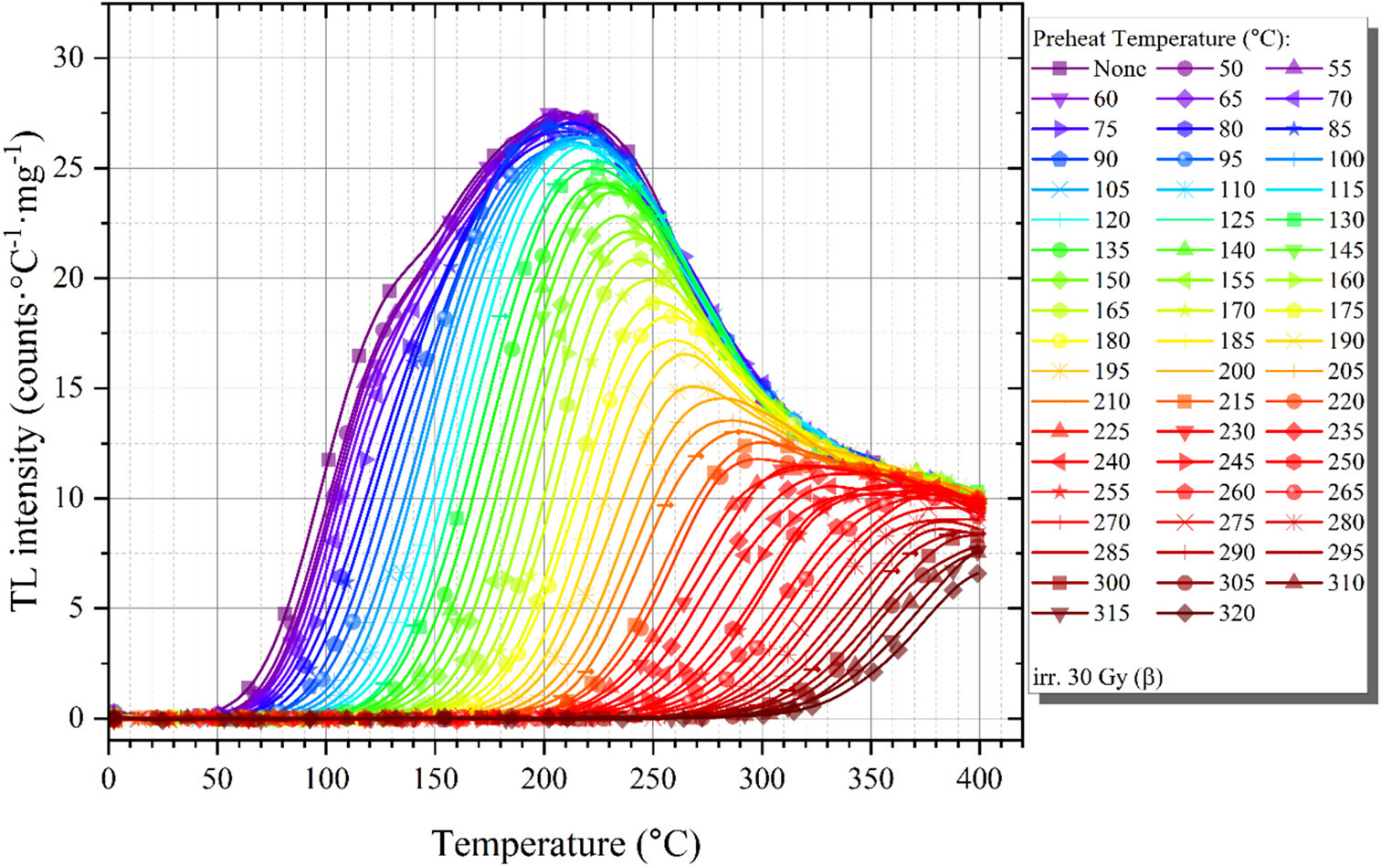
Net TL signals from the $T_{m}-T_{\text{stop}}$ experiment, recorded after 30 Gy of beta radiation.

**FIGURE 10:**
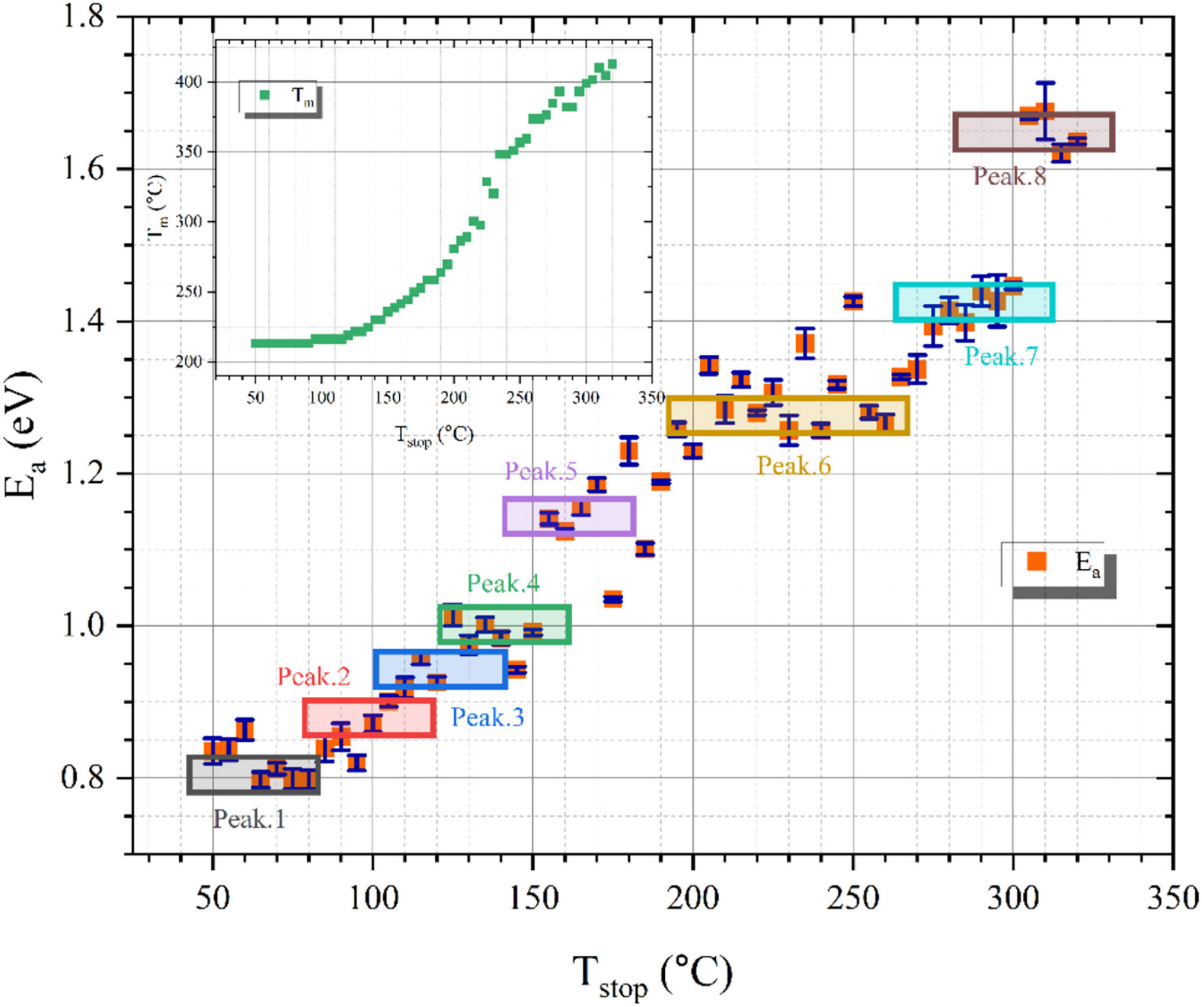
Plot of $E_{a}$ vs. $T_{\text{stop}}$ obtained using IR method and $T_{m}-T_{\text{stop}}$ as inset.

In this section, the initial rise method was used to evaluate the activation energy of glow peaks in obsidian. A method for scrutinizing the energy levels that comprise the TL glow curve involves the repeated measurement of the TL signal subsequent to a series of TL readings following thermal cleansing [23]. To perform a comprehensive analysis of the entire glow curve, the sample was first exposed to 30 Gy beta irradiation. The irradiated sample was preheated to 50°C with a heating rate of 5°C/s and then rapidly cooled to room temperature. After this irradiation, preheating, and cooling to room temperature, TL reading was performed for the glow curve at a heating rate of 5°C/s. This procedure was executed multiple times, with the identical specimen being subjected to re‐irradiation repeatedly. At the same time, the $T_{\text{stop}}$ parameter was also increased each time to a slightly higher value. Namely, the initial preheating temperature was established at 50°C and subsequently elevated to 320°C in increments of 5°C for each cycle. Figure 9 depicts the residual net TL signals obtained following each successive instance of postirradiation thermal cleaning. It is clearly seen in this figure that the traps are gradually emptied with each thermal cleaning step. As suggested in the literature, the activation energies of individual components were determined by the initial rise technique, which involves analyzing the first 5%–15% of the TL maximum intensity on the lower temperature side of the TL spectrum [46]. A graphical representation of $\ln\left(I\right)$ as a function of 1/kT produces a linear correlation that yields a slope equivalent to $E_{a}$, which can be determined by analyzing the data from the initial segment of TL glow curves, extending up to 15% of the peak maximum ($I_{m}$) [47]. In this context, $E_{a}$ denotes the activation energy, k represents the Boltzmann constant, and the temperature is expressed in Kelvin (K). The $T_{m}-T_{\text{stop}}$ graph and the activation energy $E_{a}$‐$T_{\text{stop}}$ graph obtained as a result of the applied initial rise technique are presented in Figure 10. Energy distribution reflects the activation energy levels of the individual components that constitute the composite structure of the TL glow curve. In this study, at least eight activation energy levels, ranging from 0.80 to 1.67 eV, are proposed based on the experimental results. These findings provide evidence that the TL glow curve of the obsidian sample is composite, despite the presence of primarily one prominent TL maximum when considering the entire glow curve.

To conduct an examination of the intricate TL glow curves of obsidian specimens, the CGCD methodology was utilized to disentangle the superimposed peaks and to derive their kinetic parameters. This methodology offers a robust and reproducible curve fitting approach, attaining optimal concordance between theoretical predictions and empirical observations through iterative adjustments of the model parameters. Insights derived from the $T_{m}-T_{\text{stop}}$ analysis were instrumental in this process, providing essential information regarding the number and approximate locations of the contributing TL peaks. Such knowledge significantly improved the efficiency and accuracy of the deconvolution procedure. The deconvolution analysis was performed using the open‐source R package “tgcd,” thereby ensuring both flexibility and reproducibility within the computational workflow [48]. A CGCD analysis was performed using the equation provided in Equation (4) [49].

(4) $$\begin{array}{c} I\left(T\right)=I_{m}\exp\left(\frac{E_{a}}{kT_{m}^{2}}\left(T-T_{m}\right)\right){\left[\frac{1}{b}+\frac{b-1}{b}\exp\left(\frac{E_{a}}{kT_{m}^{2}}\left(T-T_{m}\right)\right)\right]}^{-\frac{b}{b-1}} \end{array}$$

Here, $I_{m}$ indicates the TL intensity maximum, $T_{m}$ is the corresponding temperature, $E_{a}$ is the activation energy, k is the Boltzmann constant, and b is the kinetic order. These parameters characterize the trapping and recombination processes that are essential to TL phenomena.

**FIGURE 11:**
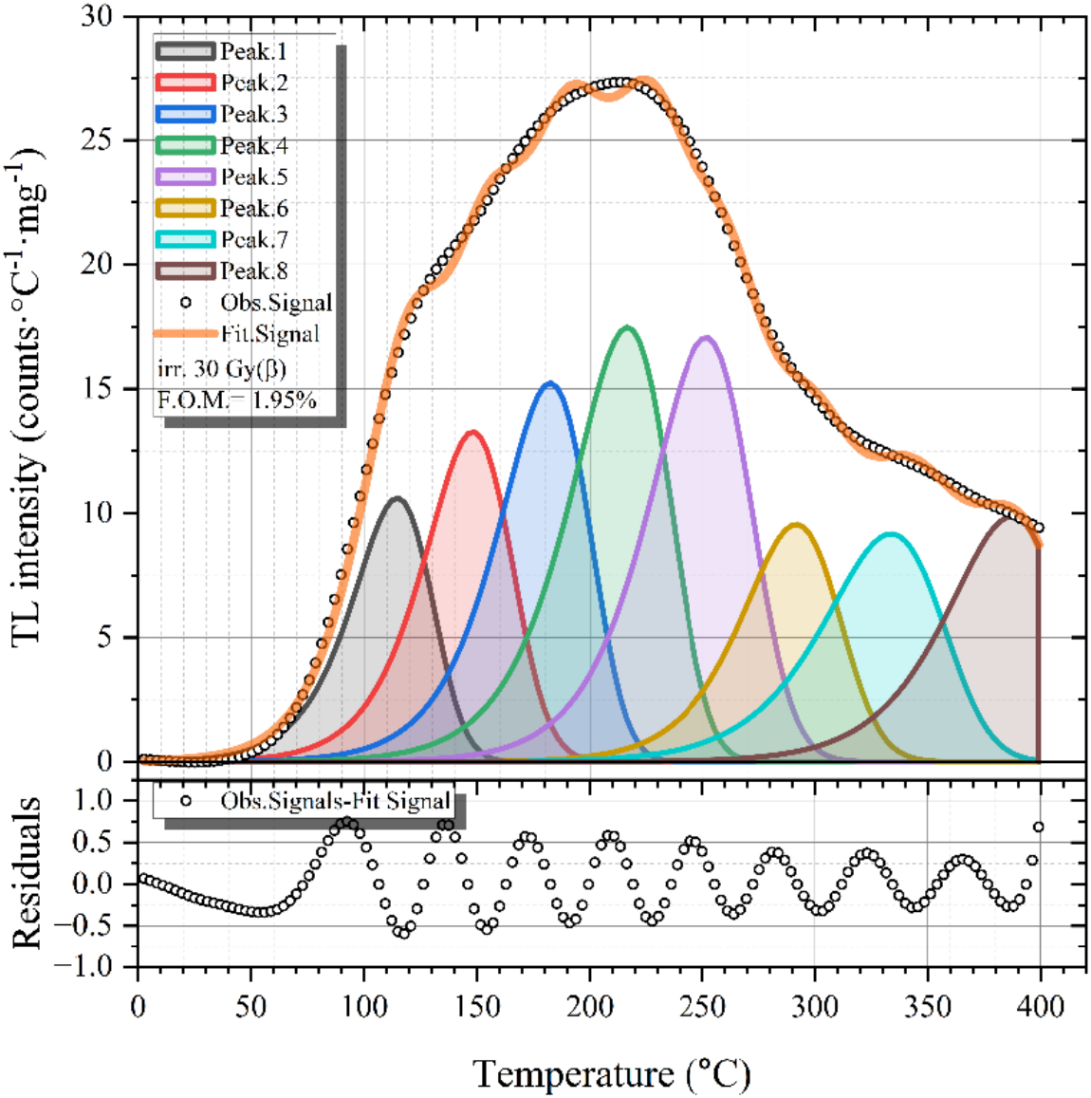
CGCD analysis of obsidian sample.

The resultant deconvolution is illustrated in Figure 11, where the experimentally obtained net TL signal of the obsidian sample (following exposure to 30Gy of beta radiation) is compared with the theoretical signal. This theoretical signal represents the cumulative sum of the TL peaks obtained from the fitting process. The fitting results are given as acceptable values in the range of 0%–2.5% [50, 51]. The obtained result was determined as a value of 1.95% (F.O.M.), which is within the acceptable range. Table 3 indicates the parameters associated with the TL glow peaks, which were derived from the deconvolution process applied to the TL glow curve. The activation energy ($E_{a}$), the temperature at which the maximum peak occurs ($T_{m}$), the kinetic order (b), the symmetry factor (μ), and the frequency factor (s) have been documented for eight distinct glow peaks, providing an extensive understanding of the characteristics associated with trapping and recombination processes. Table 3 also presents the lifetimes calculated using Equation (5), in which the temperature (*T*) is assumed to correspond to room temperature [34].

(5) $$\tau \left(T\right)=\frac{1}{s\;\exp\left(-\frac{E_{a}}{k.T}\right)}$$

**TABLE 3:** TL glow peak parameters obtained from the deconvolution analysis of the TL glow curve for obsidian.

Peaks	*E*_*a*_ (eV)	*T*_*m*_ (°C)	*b*	*μ*	*s* (s^−1^)	*τ*
First peak	0.82	114.63	1.2	0.42	1.40 × 10^10^	2.49 h
Second peak	0.90	148.06	1.25	0.42	1.74 × 10^10^	47.50 h
Third peak	0.96	182.32	1.14	0.42	1.06 × 10^10^	34.90 days
Fourth peak	1.01	216.44	1.12	0.42	6.67 × 10^9^	402.00 days
Fifth peak	1.19	251.62	1.22	0.41	6.51 × 10^10^	5.12 × 10^4^ days
Sixth peak	1.34	291.65	1.24	0.42	2.09 × 10^12^	1.66 × 10^3^ years
Seventh peak	1.45	333.60	1.18	0.41	2.93 × 10^10^	9.19 × 10^6^ years
Eighth peak	1.68	387.03	1.24	NA	1.36 × 10^12^	1.78 × 10^9^ years

The activation energy ($E_{a}$) values range from 0.82 eV for the first peak to 1.68 eV for the eighth peak, indicating a progressive increase in trap depths with ascending peak temperatures. Correspondingly, the peak temperatures ($T_{m}$) span from ~114°C to ~387°C, reflecting the sequential thermal release of charge carriers from traps of varying depths. The kinetic order (b) values, primarily ranging from 1.12 to 1.25, suggest behavior close to first‐order kinetics, although the general order kinetics equation was applied. The frequency factor (s) values range from 10^9^ to 10^12^ s^−1^. Additionally, the symmetry factor (μ) values, which range from 0.41 to 0.42, confirm that the glow peaks are well‐resolved and symmetric, consistent with the characteristics of first‐order kinetics. The calculated trap lifetimes vary across a wide range, supporting both short‐ and long‐term radiation storage. Shallow traps (0.82–0.90 eV) exhibit short lifetimes, such as τ=2.49 h and τ=47.50 h, releasing charge carriers at low temperatures but remaining thermally unstable under ambient conditions. Despite their limited storage capacity, these shallow traps are advantageous for applications requiring rapid signal readout, such as real‐time radiation monitoring or low‐dose detection. In contrast, medium and deep traps display significantly higher activation energies and exceptionally long lifetimes, extending from thousands to tens of thousands of days. For instance, the fifth trap (*E*_*a*_ = 1.19 eV) possesses a lifetime of $\tau =5.12\times 10^{4}$ days. These traps ensure the stability and reliability of stored radiation information over many years, making them critical for archival dosimetry and retrospective analysis of radiation exposure, where long‐term data preservation is of great importance. The coexistence of short‐ and long‐lived traps highlights the material's versatility, enabling it to meet a broad range of dosimetric requirements. While shallow traps facilitate immediate feedback, deep traps provide reliable and long‐term storage of radiation‐induced charge carriers.

Overall, the experimentally observed values show strong agreement with the theoretical solutions presented in this study.

## 4 Conclusions

In this study, the TL properties of obsidian samples from Aksaray region were investigated under beta irradiation, with particular emphasis on the effect of grain size. For the effect of particle size, TL measurements were taken for all particle sizes in the range of 63–500 μm, the fraction between 63 and 125 μm exhibited the highest sensitivity; therefore, all subsequent TL measurements were conducted using this size range. The reliability of the responses exhibited by the selected obsidian samples (63–125 μm) was confirmed through the conduct of a reproducibility investigation. Reusability tests demonstrated minimal signal variation, thereby affirming the stability of the material for repeated applications. The dose–response behavior of the sample displayed a stable sublinear relationship in the dose range between 0.5 and 200 Gy (*b* = 0.90675 + 0.008, *R* ^2^ = 0.99921). The LDL was determined as 71.85 ± 27 mGy. These results indicate that the obsidian sample possesses potential for environmental and personal radiation monitoring, particularly in low‐dose applications. Heating rate effects on the obsidian sample were tested using values ranging from 0.1°C to 10°C/s, and anomalous heating rate effect was observed. TL kinetic analysis was performed based on the $T_{m}-T_{\text{stop}}$ experimental data and the CGCD method. The results identified multiple TL peaks with activation energies between 0.82 and 1.68 eV, which are in good agreement with the theoretical predictions. In conclusion, this research provides a comprehensive characterization of regionally sourced natural obsidian through the combined application of the $T_{m}-T_{\text{stop}}$ and CGCD methods. The findings enhance understanding of the trap structure and TL behavior of natural obsidian and underscore its potential significance for radiation dosimetry across a range of practical applications.

## Author Contributions

**Hamide Avci:** conceptualization, investigation, methodology, validation, data curation, supervision, writing – review and editing, writing – original draft, resources, formal analysis, visualization.

## Funding

The author has nothing to report.

## Conflicts of Interest

The author declares no conflicts of interest.

## Data Availability

The data that support the findings of this study are available on request from the corresponding author. The data are not publicly available due to privacy or ethical restrictions.
